# Immunohistochemical expression of IL-1β, IL-6, and NF-κβ in fibroids

**DOI:** 10.3389/fimmu.2025.1571585

**Published:** 2025-07-02

**Authors:** Mateusz de Mezer, Natalia Dolata, Janina Markowska, Monika Krzyżaniak, Agnieszka Naskręt-Grochowalska, Jakub Żurawski, Anna Markowska

**Affiliations:** ^1^ Department of Immunobiology, Poznan University of Medical Sciences, Poznan, Poland; ^2^ Gynecological Center, Poznan, Poland; ^3^ Department of Clinical Pathology and Immunology, Poznan University of Medical Sciences, Poznan, Poland; ^4^ Department of Oncological Pathology, Lord’s Transfiguration Clinical Hospital, Partner of Poznan University of Medical Sciences, Poznan, Poland; ^5^ SPZOZ in Gostyn, Gostyn, Poland; ^6^ Department of Perinatology and Women’s Diseases, Poznan University of Medical Sciences, Poznan, Poland

**Keywords:** uterine fibroids, IL-1β (Interleukin-1 beta), IL-6 (Interleukin 6), Nf-κβ, myoma

## Abstract

Uterine fibroids are benign tumors that occur in a large proportion of women and interfere with the proper functioning of this organ. One of the factors leading to these proliferative changes appears to be the appearance of extracellular matrix (ECM) fibrosis at the site of local inflammatory foci. Due to the potential impact of cytokines in this process, it is interesting to determine their expression levels in fibroids and surrounding tissues, which may contribute to a better understanding of the mechanisms leading to the formation of these tumors. In tissue material from 50 women with uterine fibroids who underwent hysterectomy and 45 women operated on for other reasons (most often prolapse of the reproductive organ), the concentration of inflammatory cytokines IL-1β and IL-6 and the concentration of the transcription nuclear factor NF-κβ were determined. The tissue from the fibroid, the peripheral myometrium, and the unchanged myometrium were examined in women who underwent surgery for reasons other than uterine fibroids. A significant decrease in IL-1β levels was observed in the center of the fibroid compared to both peripheral and control muscle tissue (p=0.001). The concentrations of IL-6 were found to be similar across all three locations examined. The NF-κβ levels were significantly lower in the fibroid and peripheral tissues (p<0.001) compared to the control group. The concentration of IL-1β was found to be significantly and positively correlated with the concentration of NF-κβ in uterine fibroids.

## Introduction

Uterine fibroids are the most common benign tumors of the uterus, primarily composed of smooth muscle and extracellular matrix. These conditions affect about 50-70% of women of reproductive age (1–3). This varied frequency depends on several factors, including detection methods, the studied population (fibroids are found in approximately 80% of Black women), women’s age, ovarian steroid hormone status -estrogens and progesterone - genetic aberrations, and lifestyle choices (1–5).

More than 50% of women with fibroids do not experience any symptoms, and these growths are often discovered by chance. However, for some women, fibroids can lead to issues such as heavy menstrual periods, uterine bleeding, pelvic pain, infertility, complications during pregnancy and childbirth, and urinary problems. These symptoms require medical attention and can significantly impact a woman’s quality of life (1, 2, 6, 7).

The development of fibroids is influenced by various factors, including chromosomal aberrations, ovarian steroid hormones, inflammatory cytokines, growth factors, microRNA, the extracellular matrix (ECM), and stem cells (3, 8–11).

Genetic changes are an established factor in the development of fibroids. The most common mutations occur in the *MED12* gene in 50% to 80% of fibroids. This mutation is linked to a positive hormonal response to selective progesterone receptor modulators (SPRM) treatment. Other less common genetic alterations include overexpression of *HMGA2* (high mobility group protein), which is seen in 10% to 20% of fibroids, deficiencies in fumarate hydratase (*FH*), and deletions in *COL4A5/A6* that are associated with collagen formation (1, 5, 9).

Studies indicate that inflammation significantly contributes to the development of uterine fibroids (8, 12–14). In uterine fibroids, proinflammatory cytokines are overexpressed (3, 12–14). Proinflammatory cytokines include: IL-1, IL-6, IL-10, TNFα and TNFβ. Cytokines are small proteins released by various cell types that serve as intercellular messengers. They bind to specific receptors on target cells, transmitting signals that activate numerous genes with the involvement of transcription factors from the nuclear factor family, such as NF-κβ. This process regulates cell proliferation, survival, and apoptosis. Additionally, cytokines play a significant role in the interactions between growth factors and the extracellular matrix (ECM). This interaction may lead to excessive ECM production, a characteristic feature of fibroids crucial for their growth (9, 13, 15, 16).

Interleukin 1β (IL-1β) is a pro-inflammatory cytokine primarily secreted by macrophages; its overexpression is associated with various inflammatory diseases, such as osteomyelitis. Its presence has been detected in uterine fibroids, which play a role in the accumulation of ECM, a characteristic feature of fibroids. Additionally, IL-1β activates mesenchymal stem cells and the NF-κβ pathway, which are linked to the development of fibroids (12, 14, 17, 18). Interleukin 6 (IL-6) is a pleiotropic cytokine of key biological importance for regulating inflammatory processes and the immune response in various physiological states (19). Monocytes and macrophages primarily secrete it, although other cells, such as fibroblasts, produce it. IL-6 functions by binding to specific receptors. Its role can be either pro-inflammatory or anti-inflammatory, demonstrating bidirectional activity. For instance, the liver’s metabolism has shown both anti-inflammatory and carcinogenic effects. Additionally, research has highlighted its involvement in various pathways, including STAT3 (20, 21). Compared to healthy individuals, an extensive meta-analysis of 22 studies involving over 5,200 patients with various diseases demonstrated that serum IL-6 concentrations are significantly higher in sick individuals than in the control group (22).

Nuclear factor kappa Beta (NF-κβ) is a transcription factor primarily found in the cytoplasm of cells. Upon activation, it translocates to the nucleus and regulates the expression of numerous genes. NF-κβ plays a crucial role in regulating the inflammatory response involved in the development of uterine fibroids, as well as many malignancies and chronic diseases (11, 14, 23, 24). Its activity has also been demonstrated in uterine fibroids. The NF-κβ pathway can be triggered by various stimuli, including proinflammatory cytokines involved in ECM accumulation, angiogenesis, and modulation of stem cell expression. *In vivo* studies have demonstrated that inhibiting the canonical NF-κβ pathway reduces the expression of pro-inflammatory cytokines, such as IL-6 and IL-1β, leading to a decrease in fibroid size (14, 22).

The decreased tissue expression of SERPINA3 protein in uterine fibrosis is contrary to the increase in its level observed in solid cancer tumors in previous studies (25). It can be assumed that there is a mechanism responsible for inhibiting the expression of SERPINA3 in the development of fibroids dependent on the NF-kb pathway and the cytokines IL1-βand IL-6, which may cause stimulation of tumor growth. Its precise description may enable the development of fibroid therapy.

Given the global statistics indicating a constantly increasing incidence of fibroids worldwide (26), further studies are essential and may be helpful in the prevention or therapy of these benign tumors. For this reason, we undertook studies on the differences in the levels of IL-1β, IL-6, and NF-κβ proteins in fibroids and the tissue surrounding them, compared to uterine tissue, in which such changes were not found.

## Materials and methods

### Study group

The study was conducted in accordance with the Declaration of Helsinki guidelines and received approval from the Bioethics Committee of Karol Marcinkowski University of Medical Sciences in Poznań. According to a statement from the Bioethics Committee dated January 16, 2020, the study does not meet the criteria for a medical experiment. Therefore, under Polish law and Good Clinical Practice (GCP), it is not required to undergo assessment by the Bioethics Committee. Informed consent was obtained from all participants involved in the study.

The study group comprised tissue samples from patients diagnosed with uterine fibroids through histopathological examination. It included 50 women aged between 24 and 82, with an average age of 50. The fibroids varied in diameter from 1 cm to 10 cm, with an average largest dimension of 4.3 cm.

The control group comprised tissue samples that appeared morphologically unchanged, taken from 45 women who did not have fibroids, as confirmed by histopathological examination. These women underwent surgery for genital prolapse and were aged between 56 and 69 years, with an average age of 61 years.

The patients included in our study comprised two groups: those diagnosed with fibroids and a control group consisting of individuals with genital prolapse. Importantly, none of the participants had any chronic diseases. Each patient was evaluated and qualified for surgery by an internist and an anesthesiologist. Additionally, we did not identify any diseases that would serve as risk factors, such as obesity related to endometrial cancer. As a result, we did not include a table with clinical data or perform a correlation analysis with the expression of the proteins we studied. Consequently, only the age of the patients was considered in our analysis.

### Antibodies

Protein detection was performed using the following antibodies against IL1β (Biobryt Ltd. #orb97387), IL6 (Biobryt Ltd. #orb499747), NF-κβ (Cell Signaling Technology #8242).

### Preparation of microscopic slides

The study was conducted on tissue samples arranged in Tissue Micro Array (TMA) blocks, prepared according to the previously described procedure (27), using 50 uterine fibroids and 50 adjacent uterine tissues (tumor margin) identified by a pathologist. Each fragment contained elongated smooth muscle cells without atypia and exhibited a low mitotic rate of less than 1 mitosis per 10 high-power fields (HPF). Upon microscopic examination, all analyzed tissue fragments revealed smooth muscle cells that showed no atypical characteristics and did not display significant morphological changes compared to normal myometrium, peripheral tumor tissue, and tumor cells. There was no evidence of necrosis or other degenerative changes. The only notable microscopic alteration was the distorted architecture of the tumor tissue. TMAs were assembled using the UNITMA Quick-Ray^®^ Manual Tissue Microarrayer. Each TMA contained 14 patient tissue fragments and 2 control fragments. The tissue cores were 5.0 mm in diameter. Sections intended for histopathological diagnosis preceding the described study were stained with hematoxylin and eosin. Each microarray also contained a fragment of normal uterine tissue from the control group.

### Immunohistochemical examination

Immunohistochemical examination was conducted following the procedure outlined by the manufacturer, Vector Laboratories. Tissue microarrays were deparaffinized and then rehydrated with xylene and an alcohol series. Antigens were exposed at 96°C for 20 minutes in Vector^®^ citrate-based antigen unmasking solution, pH 6.0 (H-3300). Endogenous peroxidase activity was quenched in BLOXALL blocking solution for 10 minutes.

Nonspecific binding was blocked using 2.5% normal horse serum from the ImmPRESS^®^ Horse Anti-Rabbit IgG PLUS Polymer Kit Peroxidase (Vector Laboratories, CA, USA, MP-7801) for 20 minutes. After this, the excess serum was removed from the sections. Next, the slides were incubated at 37°C for 30 minutes with antibodies targeting the proteins of interest. Following this incubation, the slides were washed in PBS buffer for 5 minutes and then treated for 30 minutes with the ImmPRESS reagent. The sections were then washed twice for 5 min in PBS buffer. After removing the buffer from the sections, they were incubated in the ImmPACT DAB EqV working solution until the desired color was obtained. They were washed twice for 5 minutes in PBS buffer. The slides were then stained with hematoxylin. Finally, the sections were dehydrated in alcohol solutions and xylene before being covered with coverslips.

In the immunohistochemical reactions, tissue samples from typical uterine fragments were used as positive controls. The tissue material underwent the same staining procedure for negative controls but without the original antibody. Additionally, utilizing a tissue microarray (TMA) containing various biological samples and control tissues allowed for observing diverse immunohistochemical images within each tissue set. This created a control system that evaluated the accuracy of the immunohistochemical reactions.

A specialist pathologist assessed the differences between the myoma at its center and periphery and the surrounding normal uterine tissue. According to the pathologist’s evaluation, no necrosis features were observed in the analyzed samples.

### Semi-quantitative assessment of IL1β, IL6 and NF-κβ proteins expression

For each patient, 10 images of the field of view were captured at a total magnification of 400x using an Olympus Grundium Ocus 40 microscopic scanner (Olympus, Tokyo, Japan). A semi-quantitative assessment of the immunohistochemical reactions was conducted based on the photographic documentation, utilizing the commercial Olympus cellSens dimensional program. This program performed a phase analysis of the stained preparation by automatically detecting objects based on their color (using the brown chromogen DAB-3.3) (27).

Threshold values were established, allowing the software to classify the data automatically. During the preparation stage, both the number of cells and the area of the immunohistochemical reactions were evaluated, with the area values expressed in square micrometers (μm²). The measurement results were automatically exported to MS Excel sheets for further statistical analysis. Analyses were conducted using R version 4.2.1 (R Core Team, 2022). The software, developed by the R Foundation for Statistical Computing in Vienna, Austria, facilitated the analysis process. The mean protein level was determined for each sample as the median derived from the immunohistochemistry (IHC) signal area analysis across 10 acquired images.

To assess the normality of the distribution, the Shapiro-Wilk test was employed, which involved evaluating skewness and kurtosis values and visually inspecting the histograms. For comparisons between dependent groups, the Wilcoxon test was used, while the Mann-Whitney U test was applied for independent groups. Additionally, median differences and their 95% confidence intervals were calculated. Correlation analysis utilized the Spearman coefficient, and the significance level was established at α = 0.05.

## Results

**Figure 1:**
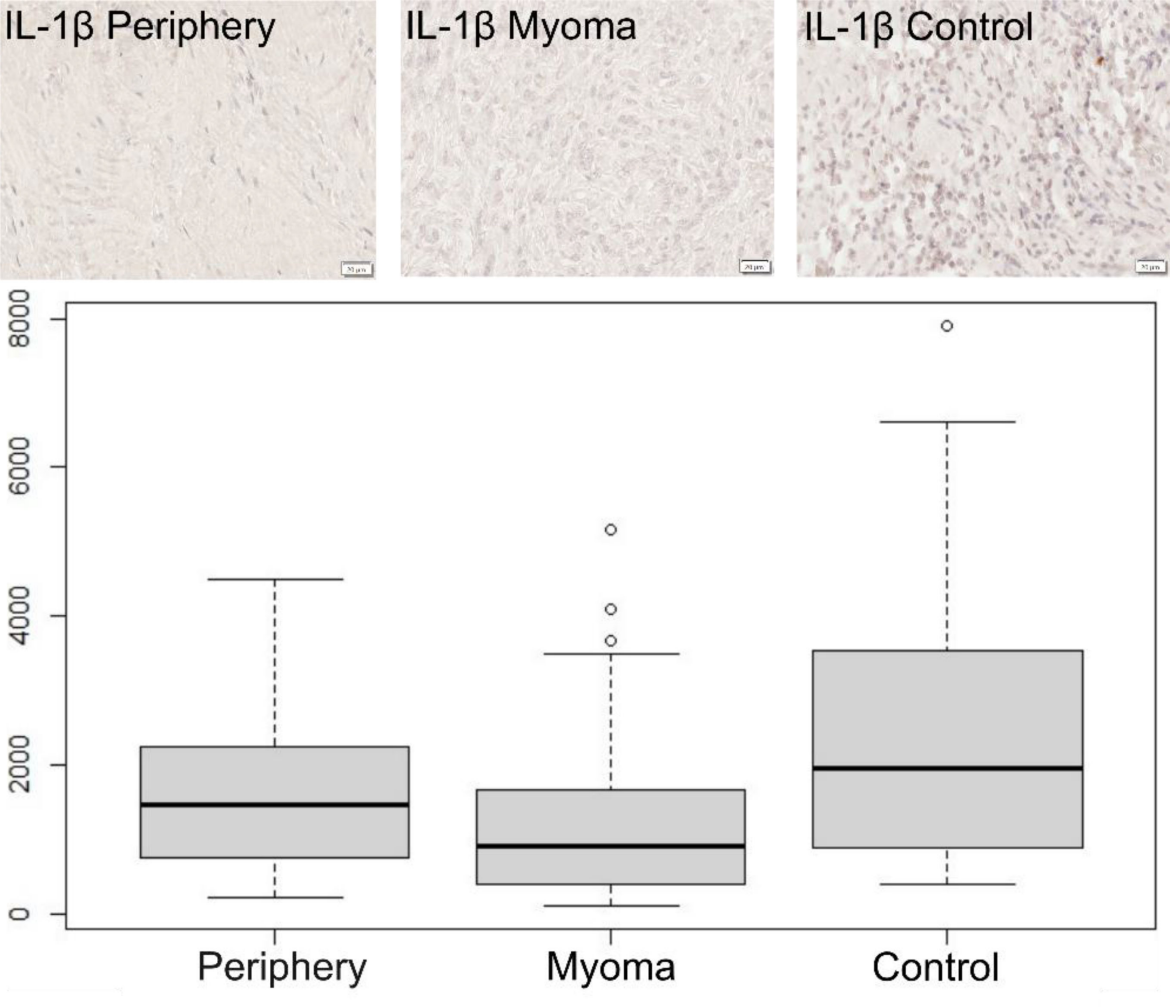
Immunohistochemical expression of IL-1β in the patient’s uterus without myoma (control) and with myoma (myoma and periphery). Magnification 400X. Boxplot of IL-1β in periphery, center, and controls. Circles indicate outlier values.

IL-1β level was significantly lower in the center of the myoma than in controls (morphologically unchanged uterine tissue), MD = -1044.49 CI95 [-1345.02; -269.91], p = 0.001 (**Table 1**). No significant differences were confirmed for IL-1β in the periphery (uterine tissue adjacent to the myoma) vs. the center or in the periphery vs. controls. These observations mean that the level of IL-1β in healthy tissues (controls) is higher than in tissues where myomas occur, while in the myomas themselves, it is lower than in the surrounding tissue (**Figure 1**).

**Table 1:** Groups’ comparison.

Variable	Group median (Q_1_; Q_3_)			Periphery vs. Center		Periphery vs. controls		Center vs. controls	
	Periphery n = 50	Centre n = 50	Control n = 45	MD 95% CI	p	MD 95% CI	p	MD 95% CI	p
IL1β	1477.76 (750.20; 2218.01)	912.56 (408.01; 1669.85)	1957.05 (897.52; 3538.99)	565.20 (-18.23; 593.22)	0.064	-479.29 (-1043.34; 21.03)	0.065	-1044.49 (-1345.02; -269.91)	**0.001**
IL6	75.68 (17.77; 161.74)	35.53 (4.84; 165.97)	28.22 (6.75; 108.89)	40.15 (-15.38; 42.42)	0.415	47.46 (-2.78; 55.30)	0.087	7.32 (-13.93; 24.61)	0.867
NF-κβ	387.40 (116.32; 670.15)	328.35 (72.51; 567.98)	2015.94 (256.88; 3324.89)	59.04 (-89.97; 251.57)	0.288	-1628.54 (-2408.79; -818.07)	**<0.001**	-1687.58 (-2473.93; -955.65)	**<0.001**

CI – confidence interval, Q_1_ – lower quartile, Q_3_ – upper quartile, MD – the median difference between groups with 95% confidence intervals (respectively: periphery minus center, periphery minus controls, center minus controls). Dependent comparisons (periphery vs. center) were made with Wilcoxon’s test, and independent comparisons (periphery vs. controls, center vs. controls) were made with Mann-Whitney’s U test.

Bold values indicate statistical significance.

**Figure 2:**
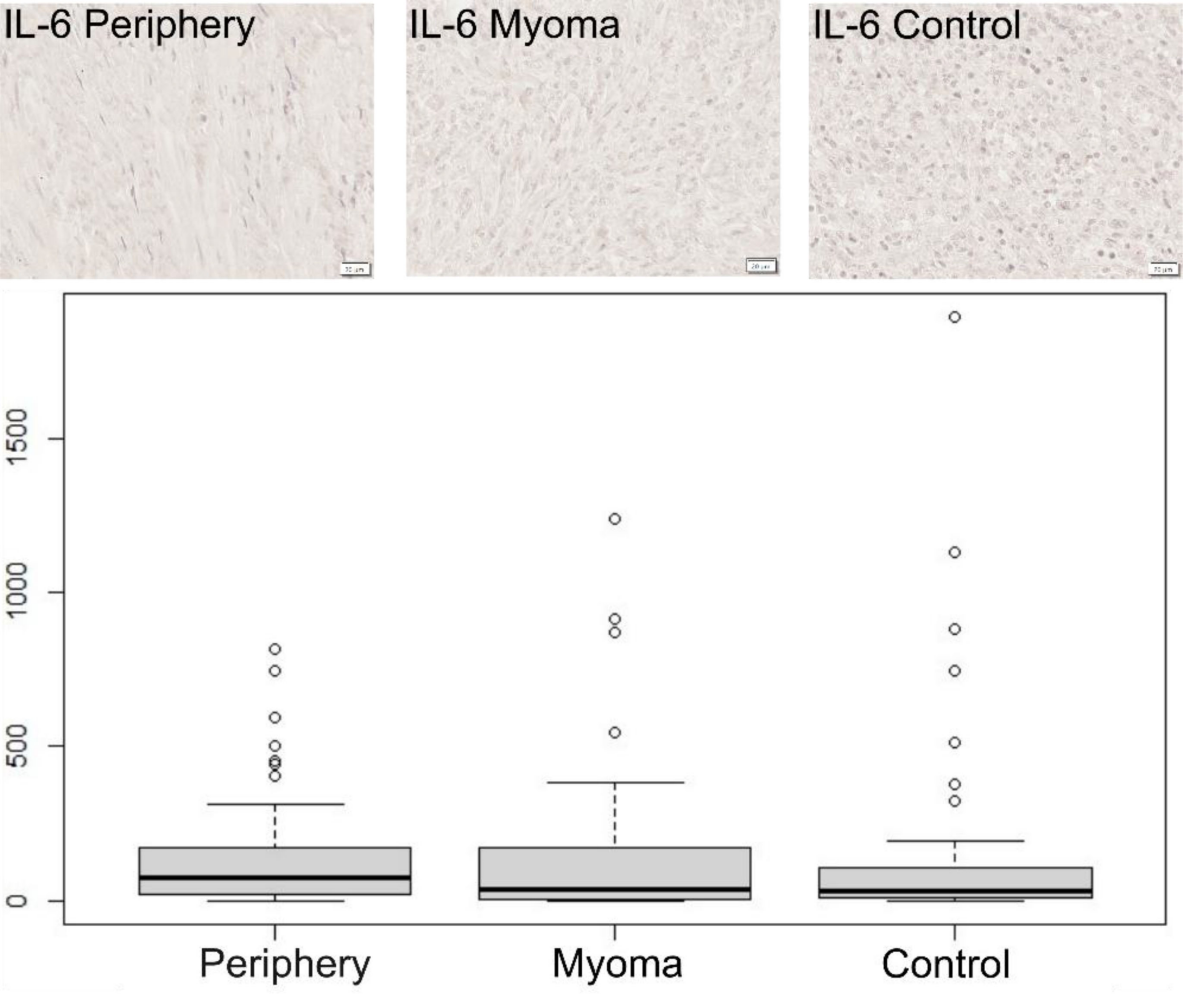
Immunohistochemical expression of IL-6 in the patient’s uterus without myoma (control) and with myoma (myoma and periphery). Magnification 400X. Boxplot of IL-6 in periphery, center, and controls. Circles indicate outlier values.

In the case of IL-6, no significant differences were found between both locations or in comparison with the control group (**Figure 2**).

**Figure 3:**
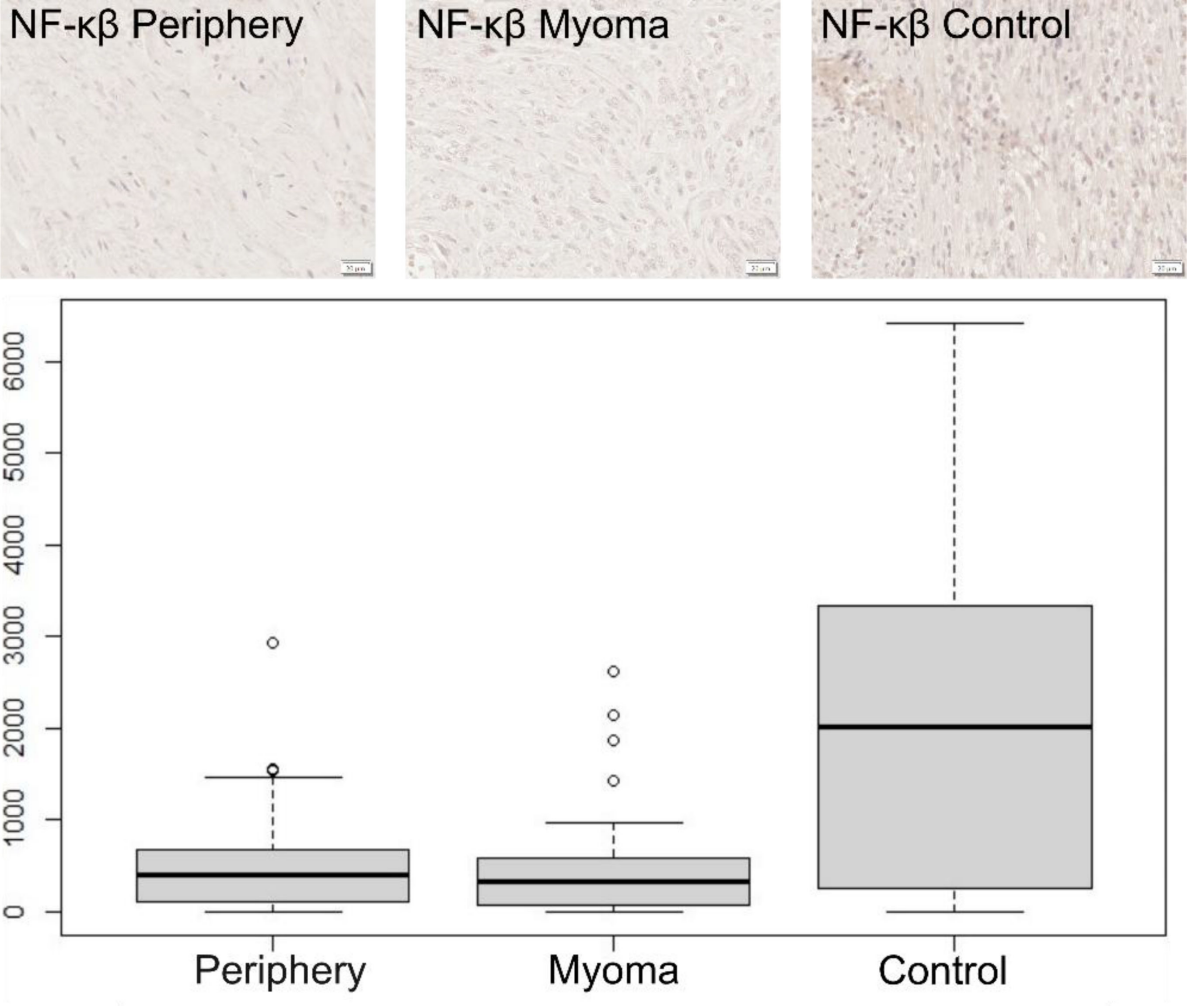
Immunohistochemical expression of NF-κβ in the patient’s uterus without myoma (control) and with myoma (myoma and periphery).Magnification 400X. Boxplot of NF-κβ in periphery, center, and controls. Circles indicate outlier values.

In the case of NF-κβ, no significant difference was observed between the tissue of the periphery and myomas (**Figure 3**). However, the level of NF-κβ in tissues where myomas were present, both in the center and in the periphery, was significantly lower than in the control group, MD = -1628.54 CI95 [-2408.79; -818.07], p < 0.001 and MD = -1687.58 CI95 [-2473.93; -955.65], p < 0.001, (**Table 1**).

No significant correlation was found between SERPINA3 and all analyzed proteins: IL-1β, IL-6, NF-κβ (p > 0.05 in all cases, both for the periphery and the center), (**Supplementary Figure 1**).

IL-1β exhibited a significant positive correlation with NF-κβ in myomas (r = 0.32, p = 0.022), although the strength of this correlation was weak. No significant correlation was found between IL-1β and NF-κβ in the periphery.

IL-6 was not correlated with NF-κβ (p > 0.05 for both locations), **Table 2**.

**Table 2:** Correlation between IL-1β, IL6 and NF-κβ.

Correlation with NF-κβ:	Periphery		Centre	
	R	p	r	p
IL-1β	0.21	0.144	0.32	0.022
IL6	0.09	0.513	0.15	0.308

## Discussion

We measured the concentrations of the cytokines IL-1β and IL-6 and NF-κβ in uterine fibroids since many authors suggest these levels change in these tumors (3, 7, 12, 22). The focus of our earlier studies on uterine fibroids was the expression of the SERPINA3 protein, which is linked to both the transcription factor NF-κβ and the cytokines we identified (28). In the review by Ishikawa et al. (12), the expression of pro-inflammatory cytokines, including IL-1β and IL-6, was assessed in the endometrium of women with uterine fibroids, as well as in the tissue surrounding the fibroid, but not within the fibroid tissue itself. A significant decrease in IL-1β and IL-6 was observed in the endometrium of women with fibroids, alongside a notable increase in cyclooxygenases COX1, COX2, and VEGF. According to the authors, these changes may impair the receptivity of the endometrium during pregnancy implantation. However, it was also noted that the uterine muscle surrounding the myomas exhibited increased macrophages and heightened IL-6 activity. Therefore, the findings from these studies cannot be directly compared to ours due to the different locations of the studied factors within the uterus.

Our research found that the level of IL-1β was significantly lower in the center of the myoma compared to the surrounding tissue and the healthy control uterine muscle. Furthermore, there was a significant positive correlation between the concentration of IL-1β and reduced NF-κβ levels in the center of the myoma.

Controversial findings regarding IL-1β levels in uterine fibroids were reported by Plewka et al. (29). They observed an increase in IL-1β and NF-κβ levels in uterine fibroid tissue compared to the control myometrium in women of reproductive age and those in perimenopause. In women of reproductive age, the highest IL-1β levels were associated with larger fibroids. However, no correlation was found between NF-κβ levels and the size of the fibroids; these levels were also lower in perimenopausal women than in those of reproductive age. These somewhat conflicting results suggest that further studies are needed.

The biological function of IL-6 is multifaceted. Although it has pro-inflammatory properties, it can also inhibit inflammation (20). In our study, the IL-6 levels measured in fibroid tissue, its periphery, and in the healthy uterine muscle of control women did not differ significantly. Additionally, we found no correlation with SERPINA3 levels in the specific areas examined. Therefore, according to the results of our studies, IL-6 does not appear to play a role in the development of uterine fibroids.

The studies by Konenkov et al. (30) concerned the level of IL-6 in the serum of women with uterine fibroids. They also did not differ from the serum IL-6 concentrations in the control group. According to the review by Ishikawa et al. (12), inflammatory cytokines play a role in uterine fibroids, and it was noted that the level of IL-6 is decreased in the endometrial tissue of women with uterine fibroids. The concentration of this cytokine in the fibroid tissue was not studied.

Our study’s results indicated that the level of NF-κβ, the primary regulator of the inflammatory response, was lower in the myoma tissue and the surrounding myometrium compared to the healthy myometrium in the control group of women. Chuang and Khorram (31) investigated the expression miR-29 in the myomas of women who underwent hysterectomies. They found that miR-29 levels were reduced in myoma tissue, which correlated inversely with the expression of genes such as COL3A1, responsible for collagen production, a key component of the ECM. The authors noted that this reduction in miR-29 is associated with a significant increase in phosphorylated NF-κβ expression in uterine myomas. To further explore this connection, the researchers applied a preparation called Bay11-708L-phenylvinylsulfane to myoma xenografts *in vivo*. This treatment effectively decreased NF-κβ concentration and positively influenced the reduction of growth and progression of human myomas in a mouse xenograft model.

Kali and Cagiran (23) also studied NF-κβ concentrations in uterine fibroids. However, they measured this factor in endometrial tissue collected from women before and after myomectomy. They found that NF-κβ levels were significantly higher before myomectomy than after myomectomy. This procedure concerned fibroids of FIGO type 3 and 4 (intramural fibroids). In light of the reports presented by other authors and their comparison with our results, which precisely determine the NF-κβ concentration in clinically significant locations, further observational studies or studies combining many factors initiated by, among others, Liu et al. (32) would be indicated. They concerned the NF-κβ receptor (RANKL), progesterone receptor (PR), DNA methylation, the influence of MED12 mutation, and stem cell expression in uterine fibroids.

## Conclusions

**Figure 4:**
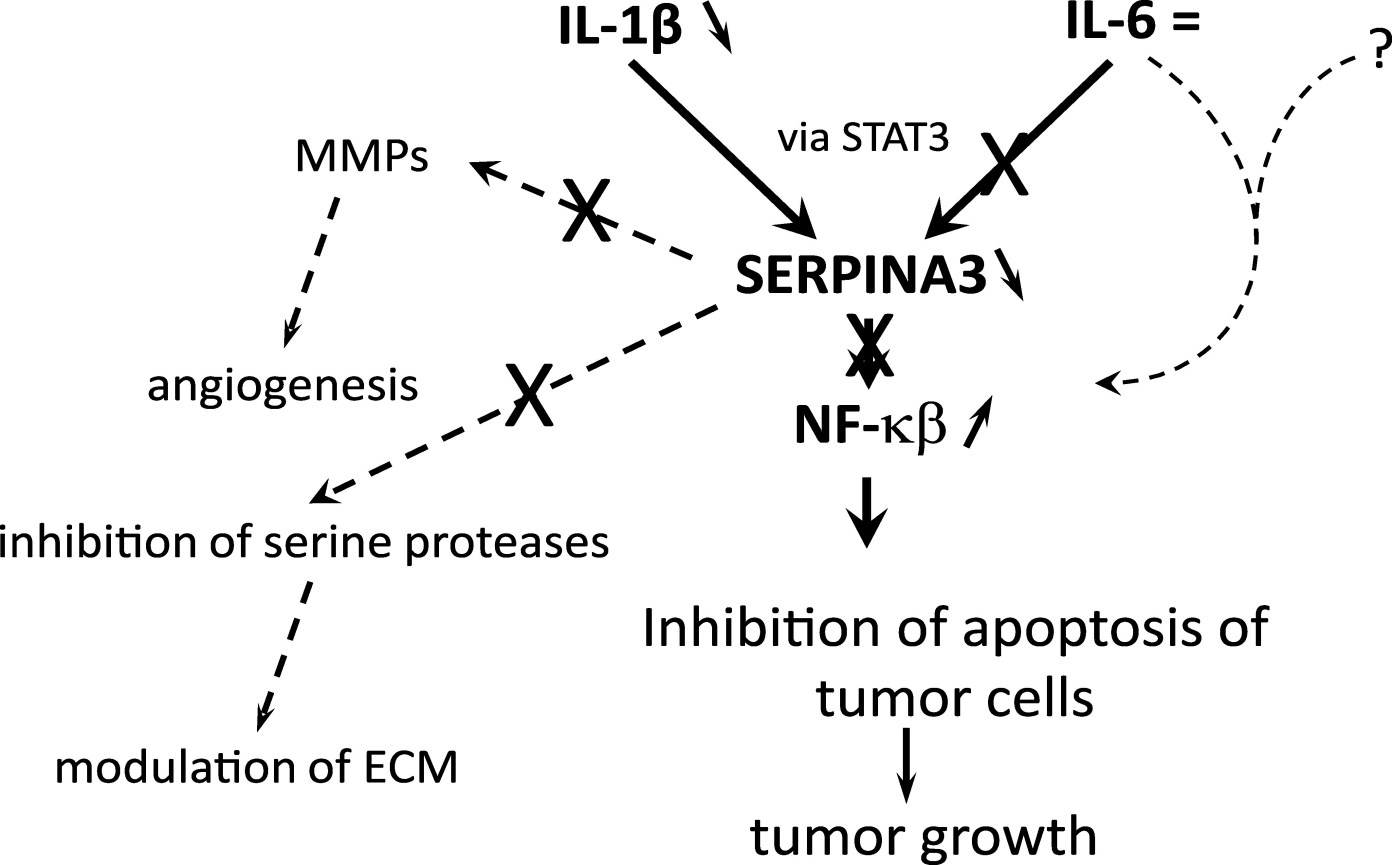
Mechanism of promotion of myoma growth by silencing inflammation inside the fibroid.

Results of our studies indicate a role for IL-1β and NF-κβ in the development of myomas. We are the first researchers to determine the levels of proinflammatory cytokines IL-1β and IL-6 and the transcription factor NF-κβ, as well as the protein SERPINA3 (28), in three locations, including one control site, who found changes in the levels of three of them involved in modulating the development of myomas. The lower level of SERPINA3 protein in fibroids (28) and the lower level of IL-1β shown in these studies confirm the inhibition of inflammatory processes in developing this type of change. However, the increased amount of the NF-κβ factor indicates that these are rather processes associated with the stimulation of cell division and the inhibition of cell death. Based on the results presented in this and previous studies (28, 33), we can propose a mechanism in which the silencing of inflammation inside the fibroid promotes its growth (**Figure 4**). Lowering IL-1β levels can inhibit the action of the transcription factor STAT3, resulting in lower SERPINA3 levels. Interestingly, IL-6 does not seem to be involved in this regulation, after which we observed no change in its level. On the other hand, a lower amount of SERPINA3 protein should lead to a decrease in the amount of NF-κβ and cell apoptosis, thus inhibiting tumor growth. However, our observation of increased expression of NF-κβ indicates that it nevertheless acts by inhibiting processes leading to cell death and enabling further tumor growth. In regulating the level of NF-κβ, another factor must be involved that remains to be identified. It should also not be forgotten that the decrease in SERPINA3 levels due to the lack of IL-1β results in an increase in serine protease activity, which increases ECM remodeling. In addition, it reduces the inhibition of matrix metalloproteinases (MMPs), which may facilitate angiogenesis (33). Therefore, further research is needed to help identify the factors that induce the development of fibroids and thus indicate possible therapeutic pathways.

## Data Availability

The raw data supporting the conclusions of this article will be made available by the authors, without undue reservation.
