# Supplementary material for: Immunohistochemical expression of IL-1β, IL-6, and NF-κβ in fibroids
Source: Front Immunol. 2025 Jul 2;16:1571585. doi: 10.3389/fimmu.2025.1571585 (PMC12263548; doi:10.3389/fimmu.2025.1571585)
Supplement: Supplementary file 1 [file DataSheet1.pdf]

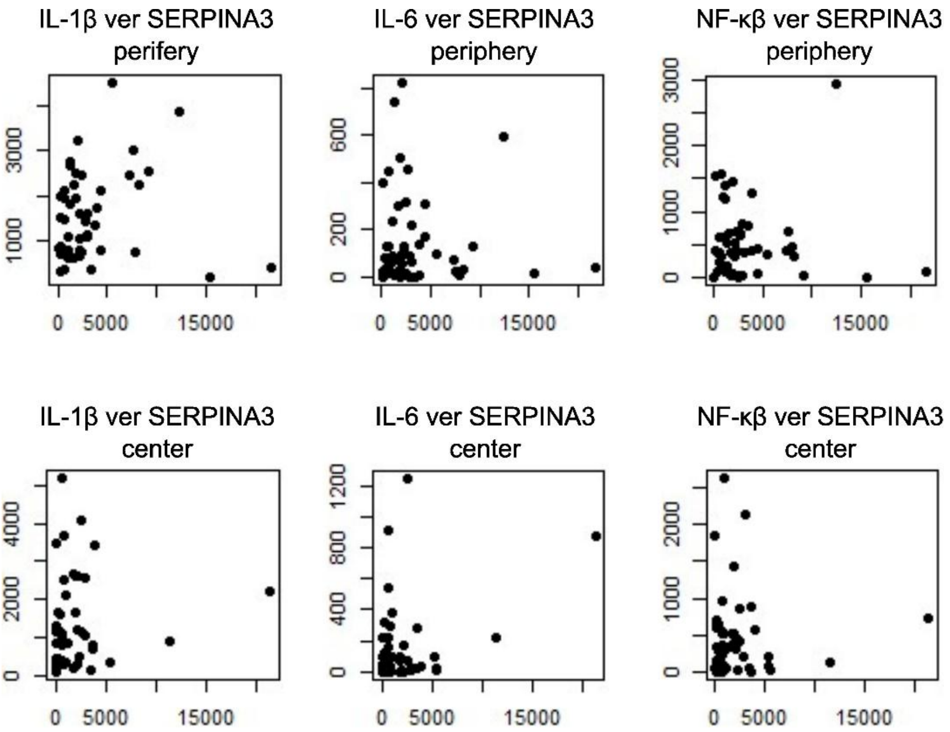

Supplementary Figure 1. Plot of IL-1β, IL6, NF-κβ versus SERPINA3 in periphery (upper panels) and center (lower panels).
